# Drivers of Palatability for Cats and Dogs—What It Means for Pet Food Development

**DOI:** 10.3390/ani13071134

**Published:** 2023-03-23

**Authors:** Pavinee E. Watson, David G. Thomas, Emma N. Bermingham, Nicola M. Schreurs, Michael E. Parker

**Affiliations:** 1 School of Agriculture and Environment, Massey University, Palmerston North 4474, New Zealand; 2 Added Value Foods & Bio-Based Products, AgResearch Te Ohu Rangahau Kai, Palmerston North 4474, New Zealand; 3 School of Food and Advanced Technology, Massey University, Palmerston North 4474, New Zealand

**Keywords:** palatability, cat, dog, acceptance, preference, pet food

## Abstract

**Simple Summary:**

The pet food industry is growing rapidly globally. Although new products continue to be developed, research into their palatability still largely uses traditional methods. Testing focuses on the amount of food consumed, but little consideration is given to why differences are observed and which ingredients are most important. This review will discuss the feeding behaviour and nutritional requirements of dogs and cats, the main types of pet foods produced currently, and the current methods used for assessing palatability. Finally, the opportunities to use better methods to develop foods that are more palatable and understand the nutritional factors responsible for driving intake are discussed.

**Abstract:**

The pet food industry is an important sector of the pet care market that is growing rapidly. Whilst the number of new and innovative products continues to rise, research and development to assess product performance follows traditional palatability methodology. Pet food palatability research focuses on the amount of food consumed through use of one-bowl and two-bowl testing, but little understanding is given to why differences are observed, particularly at a fundamental ingredient level. This review will highlight the key differences in feeding behaviour and nutritional requirements between dogs and cats. The dominant pet food formats currently available and the ingredients commonly included in pet foods are also described. The current methods used for assessing pet food palatability and their limitations are outlined. The opportunities to utilise modern analytical methods to identify complete foods that are more palatable and understand the nutritional factors responsible for driving intake are discussed.

## 1. Introduction

Over the last decade, the pet food industry has shown strong growth both globally and more locally in New Zealand. Global pet food sales reached US$114.8 billion in 2021, compared to $78.1 billion in 2011, and were projected to surpass $123.5 billion in 2022 [1]. Within the industry, cat and dog foods have the greatest market share, accounting for US$110.6 billion or more than 96% of the pet food sales made globally in 2021 [1,2]. Sales are now projected to reach US$156.9 billion globally by 2026, with cat and dog food contributing approximately US$152 billion [1].

The dominance of cat and dog food is likely due to these species being the most common household pets [3]. In New Zealand, the pet ownership rate was one of the highest in the world in 2020, with 64% of households being home to at least one companion animal [4], and increasing rates of companion animal ownership are seen globally in developed countries [5]. Growth in the industry to date can be attributed to market trends that have resulted in a major increase in the number of new and innovative products that are available to pet owners [6].

While pet food is primarily formulated to deliver complete and balanced nutrition, palatability has been identified as a crucial factor for determining the success or failure of a product in the market and the probability of repurchase [7,8,9,10].

Therefore, this review will consider the feeding behaviour and nutritional requirements of both dogs and cats before focusing on the diet selection pattern of cats. The different formats of pet food available and the ingredients commonly included in pet foods will also be assessed, and from there, the methods used for assessing pet food palatability will be reviewed, along with a discussion of the known drivers of palatability.

## 2. Nutrient Requirements of Cats and Dogs

Cats and dogs are both members of the order Carnivora. While the name implies that both are specialised meat-eaters, each species originated from different branches, with the domestic cat (Felis catus) being part of the Felidae family and the domestic dog (Canis familiaris) being part of the Canidae family. However, the nutritional requirements, feeding behaviour and food selection choices vary considerably between the two species.

### 2.1. Domestication and Feeding Behaviours

Dogs were likely the first animals to be domesticated [11]. They share a long history of co-existence with humans, with the dog’s direct wolf ancestor (Canis lupus), thought to have been utilised as guards and hunters alongside human hunter-gathers [12]. It is believed that divergence from their carnivorous wolf ancestors took place between 13,000 and 17,000 years ago, when the increased availability of human food waste associated with the move to an agricultural existence created a new ecological niche [13]. Wolves took advantage of this new niche and became more accustomed to human contact. Over time, humans became experienced at selecting for specific tameness traits in dogs and established control over proto-dog mating, ultimately resulting in the evolution of the domestic dog [12].

Compared to their carnivorous wolf ancestors, domestic dogs can consume foods of both animal and non-animal origin and are therefore classified as facultative carnivores [13,14]. They are often described as opportunistic eaters that spend a short period of time consuming large amounts of food [6,15]. As a result, food is normally eaten in a gluttonous manner with minimal chewing taking place, with food being regurgitated later when they have removed themselves from the presence of other pack members [6]. In the wild, food sources for dogs vary greatly, ranging from insects, berries, and grass through to animal faeces and carrion [6]. In addition to consuming these foods, gnawing on bones, and other animal parts is also seen in the wild dog [6]. The ability to adapt to a vast selection of foods likely resulted from needing to survive through sustained periods of feast and famine and to cope with variable nutrient availability, which in turn allowed the change from a predominantly carnivorous to a more omnivorous diet [13].

In contrast to dogs, cats were domesticated approximately 9000–10,000 years ago from the African wildcat (Felis silvestris), making them one of the most recently domesticated mammal species [12,16]. Rather than being actively sought as household pets by humans, cats likely became associated with people to take advantage of food scraps and vermin found in their settlements and are believed to have naturally diverged from their wildcat ancestors [12,17].

Cats are solitary hunters that will often kill much smaller prey than their body mass, which results in them needing to have multiple kills a day to meet their energy requirements [6]. This prey is normally eaten immediately, and cats show a preference for food at body temperature and will often consume carrion that has cooled to ambient temperatures [18,19]. Smaller prey are often consumed in one portion, but with larger prey, the flesh will be ripped off and the organs will be consumed [6]. For example, when Eurasian lynx were followed over a three-month period in spring, they preferentially ate muscle tissue, body fat, and internal organs, except the digestive tract, of 359 prey species in the wild (made up of predominately roe deer at 69%) [20]. Of these kills, the meat (muscle) and organ meats (lung, heart, kidney, liver, and spleen) were completely consumed in 90% of analysed cases [20].

Cats are also classified as intermittent feeders and consume multiple, small meals throughout a 24-h period [21,22,23]. Cats are also much more selective eaters compared to dogs and can detect small differences in the composition of the food they are offered [6,21]. Cats are defined as obligate carnivores in their methods of ingesting, digesting, and metabolising meat-based diets [21]. Without animal-derived protein, severe nutritional deficiencies can occur in cats.

Both cats and dogs tend to display neophilic behaviour, defined as liking towards a foods that is new, as opposed to neophobia, which is described as the avoidance of new food [24,25]. In extreme cases, some cats may also exhibit metaphilia, which is defined as a clear preference for change or variation from a familiar food [25]. Age also has an influence on whether an animal displays neophilia or neophobia. For example, puppies show a higher level of neophilia than adult dogs because they are constantly exploring their environment and learning (by trial and error or social learning) to eat new foods. In contrast, neophobia is an adaptive behaviour to prevent possible intoxications and displayed more in adults [24]. Overtime, the feeding experiences become less variable for cats as a dynamic equilibrium between the purchasing habits of owners and the food preferences of the cat is reached [15].

Overall, cats and dogs continue to be the most used animal models in assessing pet food palatability. While dogs show greater acceptance of a wide variety of foods, their opportunistic feeding behaviour and tendency to consume the first food chosen may prove challenging when looking to identify the fundamental components that drive food intake. In comparison, cats show greater selectivity and can detect small changes in food composition. Therefore, greater focus will be placed on the nutrient requirements of cats in this review, with reference to dogs provided for comparative purposes.

### 2.2. Protein

Despite being classified as carnivores, cats and dogs have specific dietary nutrient requirements, with cats notably having more specialised nutrient needs than dogs. For example, cats have a higher minimum requirement for dietary protein than dogs, at 26% versus 18% on a dry matter (DM) basis, with protein requirements increasing to 30% for growing stages and 22.5% for lactation [26]. Within these protein requirements, essential amino acids must also be present at specified levels to deliver a complete and balanced diet [26,27]. This is likely due to their ability to regulate the enzymes that catalyse amino acid metabolism being impaired [28]. Table 1 shows the higher minimum dietary levels of essential amino acids required by cats compared to dogs [26,29].

Taurine is the only amino acid able to conjugate bile acids in cats. Unlike other mammals that can use glycine as an alternative to taurine, cats do not possess this ability [7]. Additionally, renal function and structure are maintained by cats with the help of taurine, and it also has roles in cardiac function, sight, and reproduction. However, cats and kittens are unable to synthesise or recycle enough taurine to meet their needs, and so it must be provided in their diet.

Arginine is another essential amino acid required for growth and in the detoxification and excretion of ammonia as urea [30]. It is of great importance to cats, as ammonia toxicity can result if they are fed diets lacking in arginine [31]. Unlike other mammals that can synthesise arginine from ornithine and citrulline in the intestine, cats have a lack of the enzymes pyrroline-5-carboxylate synthase and ornithine aminotransferase, which are required to produce arginine [32].

### 2.3. Vitamins and Minerals

Cats and dogs also have requirements for vitamins and minerals, as outlined in Table 2. Briefly, the twelve essential vitamins required for adult cat maintenance are the fat-soluble vitamins: A, D, and E, and the water-soluble vitamins: thiamine, riboflavin, niacin, pantothenic acid, pyridoxine, folic acid, vitamin B12, biotin, and choline [26,27].

The macro-minerals that are essential for cats are calcium, phosphorus, magnesium, sodium, potassium, and chloride, and the microminerals that are essential for cats are iron, copper, zinc, manganese, selenium, and iodine [26,27]. In this discussion, focus will be placed on vitamin A and niacin, as these two follow unique synthesis pathways in cats. Calcium and phosphorus will also be examined as the key microminerals required in the highest amounts for the adult cat.

While the main source of dietary vitamin A for most species is in the non-toxic plant pigment form of β-carotene, cats lack the dioxygenase enzyme required to start the conversion of carotenoids to retinal, and therefore a dietary source of preformed vitamin A is required [3,7,33,34]. In contrast to β-carotene, preformed vitamin A can be toxic if consumed in large amounts [33]. As a fat-soluble vitamin, excess vitamin A is not excreted through urine when consumed in excess. Instead, appreciable amounts are stored in the liver as well as fatty tissues throughout the body [33,35]. Whilst important for vision, bone and tooth growth, as well as reproduction and maintenance of skin and mucous membranes, vitamin A toxicity in cats can result in muscle soreness, tenderness of joints, and hyperesthesia, particularly along the neck and forelimbs of cats due to the development of bony exostoses [33,35,36]. As carnivores, most of the vitamin A is consumed as preformed retinyl palmitate stored in tissue and is particularly abundant in the liver of their prey [33]. In commercial diets, a safe upper limit for vitamin A levels in cats has been set at 333,300 IU/kg, which is equivalent to 99.99 µg/g of retinol [27]. Since beef liver has been shown to have vitamin A levels of 283.19 µg/g [37], a cat would only be able to consume 35% of their total daily intake as beef liver, assuming no other ingredients supply any vitamin A.

In contrast to the synthesis pathways of taurine and arginine, which are characterised by low enzymatic activity at different points, cats possess all the enzymes and pathways required for niacin synthesis [38]. Niacin is a water-soluble vitamin essential for energy metabolism and can be metabolised in one of two ways using tryptophan; one results in the production of acetyl CoA and CO_2_, and the other to nicotinamide adenine dinucleotide (NAD) [38]. In cats, the activity of picolinic carboxylase, the enzyme catalyzing the first step of the degradative pathway to acetyl CoA and CO_2_, is upregulated, resulting in niacin being broken down faster than it is produced [39]. This upregulated catabolic pathway allows the carnivorous cat to consume a high protein tryptophan-rich diet while also preventing free tryptophan and its intermediates from accumulating to toxic levels, which can have undesirable metabolic side effects in the cat [40]. Additionally, cats are supplied with enough NAD and nicotinamide adenine dinucleotide phosphate (NADP) coenzymes through the dietary consumption of meat, meaning they have no need to produce niacin from tryptophan [38].

Calcium and phosphorus are the two most abundant minerals in the body. They are necessary for the growth and maintenance of bones and teeth, with calcium also being involved in blood clotting and nerve impulse transmission, and phosphorus playing an important role in energy metabolism as a component of adenosine triphosphate [41]. Bone material and fish provide a good amount of calcium for pet foods. Phosphorus is also provided by meat and vegetables, particularly cereal; however, in grains, phosphorus is presented in a less bioavailable form known as phytate [41,42]. In addition to these organic forms of calcium and phosphorus, inorganic sources (additives) are also used in the industry but have a higher bioavailability [43]. For example, phosphate salts are highly soluble compared to bone ingredients, resulting in increased absorption and postprandial serum levels. This can have a negative impact on phosphorus homeostasis and contribute to renal damage [41].

In addition to bioavailability, dietary levels of phosphorus and calcium can also have varying adverse effects on feline health. Low dietary levels of phosphorus are associated with an increased risk of hypercalcemia [41]. High dietary phosphorus levels greater than 3.0 or 3.6 g/1000 kcal may lead to kidney damage or dysfunction and chronic kidney disease (CKD) in healthy cats, particularly when the highly available soluble inorganic salts are provided [41,43]. High levels of phosphorus also severely disrupt the hormonal regulation of phosphate, calcium, and vitamin D [43,44]. In terms of calcium, plasma calcium levels are generally well-regulated; however, low levels can have immediate detrimental effects, including cardiac arrhythmias [41]. A sudden increase in dietary calcium may increase the risk of calcium oxalate stone formation as well as lead to calcification and possibly kidney injury and impaired function [41].

The availability of calcium and phosphorus is also impacted by their relative proportion to each other [41]. Many cases of hyperparathyroidism have resulted from a Ca:P imbalance, which can result from feeding high-protein meat products that are sufficient in phosphorus but low in calcium [35,41,42]. Conversely, increased calcium absorption may take place when presented with a high ratio of calcium to phosphorus [41]. A Ca:P ratio of 1:1 to 2:1 is considered to reduce the likelihood of calcium- or phosphorous-related issues [35].

### 2.4. Fat

Cat foods must also contain a minimum of 9% crude fat on a dry matter basis for maintenance, compared to 5.5% in dog foods [26]. Within the fat requirements, arachidonic acid is essential for cats and must be present at 0.2% on a dry matter basis in food [27]. While there are no additional fatty acid requirements for adult cats and dogs, kittens and puppies have the additional requirements for eicosapentaenoic acid (EPA) and docosahexaenoic acid (DHA). EPA is important for supporting the body’s natural anti-inflammatory response, and DHA plays a vital role in neurological and retinal development [45,46,47].

## 3. Diet Selection (Macronutrient Selection)

While the specific minimum nutrient requirements of cats and dogs have been established, research has shown that cats and dogs are able to select a ‘target intake’ of protein, fat, and carbohydrates to achieve nutritional adequacy when given the choice between diets differing in macronutrient composition [48,49].

An extensive study by Hewson-Hughes et al. [49] used geometric analysis to assess macronutrient selection in dogs when presented with six dry-format (extruded) diets and six wet-format (retorted) diets for five different dog breeds. It was found that after initially selecting a diet significantly lower in fat, dogs were able to regulate their dietary macronutrient level based on the metabolisable energy compositions of 30% protein, 63% fat, and 7% carbohydrate, with values showing similarities across the different breeds.

Prior to the work in dogs, Hewson-Hughes et al. [48] conducted the same study with cats and found that they selected dietary macronutrients based on the metabolisable energy compositions of 52% protein, 36% fat, and 12% carbohydrate. As well as the optimal levels, the study revealed that a carbohydrate ceiling effect of approximately 300 kJ/day (72 kcal/day) is displayed by cats. This low intake of carbohydrates is likely associated with many sensory and metabolic adaptations, such as their inability to detect sweetness due to their lack of sweet taste receptors [48].

When wet diets of varying protein and carbohydrate contents were fed to cats, the cats were able to regulate their macronutrient intake to obtain 53% of metabolisable energy from protein and 11% from carbohydrate [50]. The results obtained from a geometric assessment of cats’ intake of macronutrients reflect similar levels of protein, fat, and carbohydrate in prey consumed by free-roaming cats at 52:46:2% [16]. This illustrates that, in terms of macronutrient selection, cats are driven to foods with a high protein and fat content and avoid carbohydrate-rich foods.

The study by Salaun et al. [50] also assessed the effect of adding a palatability enhancer to diets and found that cats consumed more food, but no difference was observed in their protein or carbohydrate intake patterns. A follow-up study by Hewson-Hughes et al. [51] also examined the effect of adding positive (fish), neutral (rabbit), and negative (orange) flavors to diets varying in protein:fat energy ratios of 10:90, 40:60, and 70:30. Cats were able to distinguish between flavors added to the foods, with fish preferred over rabbit and no addition or flavor, and orange flavor being the least preferred in the short term. However, in the long term, cats selected similar protein and fat intake regardless of flavor combination, suggesting that macronutrient balancing is a key driver for longer-term food selection and intake in the domestic cat [51].

## 4. Types of Pet Food

Today, domestic cats and dogs receive most, if not all, of their nutritional requirements from commercially prepared pet foods. Although there are a variety of foods available, pet foods typically fall under one of three broad categories: dry, wet, or semi-moist foods, depending on their processing method, methods of preservation, and moisture content [52]. Along with these three main diet types, foods can also be formulated to be complete, balanced, or complementary. Complete and balanced foods deliver all nutrients at the correct levels to pets when fed as a single food source. In contrast, complementary foods, such as pet treats and mixers, generally lack some essential nutrients, so they can only form 10% of the daily intake and must be fed alongside another type of food to ensure the animals nutrient requirements are met [53].

### 4.1. Dry Food

Dry pet foods have a typical moisture content between 10 and 12% and rely on this low moisture content for preservation. Dry pet foods often include cereal grains and by-products, soybean products, animal by-products, fats, and oils, as well as the inclusion of vitamins and minerals, which are generally mixed to form a dough, which then undergoes further processing to extrude and dry [26,54]. There are many forms of dry pet food, including baked, air- or freeze-dried, and extruded products, with the latter accounting for most dry pet foods available on the market [26,52].

Extrusion can produce a range of products with different shapes, ss, and colours. This often has little to do with nutritional adequacy for pets but provides visual variety to pet owners [26,54].

Baked kibble and biscuits are the least common types of dry pet food. To form a dough suitable for biscuits, a formulation with a high proportion of wheat is traditionally used. For biscuits, the dough is cut into shape before baking in an oven, whereas for kibble, a large sheet is baked and then broken up to form a kibble [52,54].

Air-dried and freeze-dried pet foods are also becoming increasingly popular types of dry food on the market. Compared to traditional dry food cooking methods, air-dried pet food typically uses low drying temperatures (usually below 100 °C) with gentle airflow for a long drying time [55,56,57]. Freeze-drying is beneficial in retaining the properties of the raw material better than the air-dried product [58]. However, both options provide end products that are minimally processed to help maintain the nutritional value of the raw material, which can be lost in traditional manufacturing processes.

The ingredients included in dry pet food are much the same for cats and dogs, although more emphasis is placed on the inclusion of proteins and fats of animal origin in dry cat foods [59]. Dry pet foods have the benefit of being a relatively cheap and useful source of energy compared to wet and semi-moist pet foods. Dry foods are also very easy to store and dispense; however, they are often less palatable than the other food formats, particularly to cats [59].

### 4.2. Wet Food

Wet foods typically have a moisture content of 74–78% and exist in a variety of forms, with canned and pouch products being the most common [26,52,54]. Many of the same ingredients used in dry pet foods are also included in canned food at differing levels [26]. In canned foods, there is a much higher inclusion of fresh or frozen meat, poultry, or fish products, and animal by-products, usually at levels between 25–75%, and cereal flour is used as gelling agents [26].

**Figure 1:**
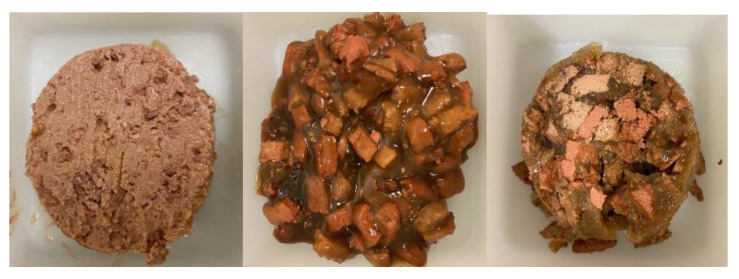
Canned wet food formats (left to right): loaf, chunks in gravy, and chunks in loaf.

There are three general types of wet food: loaf, chunks or chunks in gravy, and a chunk in loaf combination (Figure 1). All three are preserved via heat treatment, where cans are filled with the wet slurry of ingredients, sealed with a double seam lid, and retorted at a defined temperature and time profile that kills food-borne pathogens [60,61,62,63]. This produces food-safe products that have a long shelf life and no special storage considerations [26,54,59].

There can be considerable damage or loss of nutrients during heat processing and storage. During canning, ascorbic acid is unstable in the high-moisture environment of wet pet food. Heat- and moisture-liable vitamins such as thiamine, folic acid, and β-carotene also show losses. Vitamins that are usually stable, such as riboflavin, niacin, pantothenic acid, choline, vitamin B12, and biotin, have good processing resistance, except for biotin in wet dog food. Vitamin losses during storage were minimal compared to the losses during processing, due to the protective environment of the can. However, thiamine and vitamin B12 were the two main vitamins lost during storage. To combat these losses, manufacturers add compensatory amounts to formulations to ensure adequate levels are retained following heat treatment [59].

In addition to the loss of nutrients, Maillard products, formed via a chemical reaction between amino acids and reducing sugars in wet food during heating, result in the production of different flavours and a brown colour, which are associated with decreased protein digestibility but increased palatability [7,52,54,59,64]. Compared to dry foods, canned foods are generally more desired by cats as they reflect similar properties to meat and also contain little or no cereal or carbohydrate.

### 4.3. Semi-Moist Food

Semi-moist products are relatively uncommon and exhibit a moisture content which can range from 25 to 35% and are stable at room temperature [26,54]. To achieve its shelf-life stability, the water activity, defined as the water that is available for bacterial and fungal growth in or on the surface of food, needs to be controlled [59]. Manufacturers will include ingredients classified as humectants, such as salts, simple sugars, glycerol, and corn syrup, in formulations that control the water activity [26,59]. To prevent the growth of yeasts and moulds, preservatives such as potassium sorbate may also be added [52].

Semi-moist foods use similar ingredients to dry and wet foods. They are prepared in a similar manner to dry foods, with the addition of meat or meat by-products prior to extrusion. The ratio of dry to wet ingredients can range from 4:1 to 1:1 in this type of food. Semi-moist products often come out in patties or roll-like form for dogs, or in single-serve packages of small bite-sized pieces for both cats and dogs [26]. This type of food has a softer texture than dry food, which has a positive influence on food acceptance, the amount of food required to meet a pet’s caloric needs, and palatability, defined as their preference or choice for a particular food over another [52].

### 4.4. Nutritional Comparison of Different Types of Pet Food

While the three main types of pet foods have different processing methods, preservation techniques, and moisture contents, products can be compared nutritionally on a dry matter basis (Table 3).

### 4.5. Emergence of Vegetarian and Vegan Pet Food

Vegetarianism and veganism have become increasingly popular dietary choices among the global human population [65]. Vegetarians are defined as those who do not consume meat, poultry, or fish, with vegans being seen as a smaller group of vegetarians who do not consume any animal products whatsoever [66]. A study by Leahy and colleagues [67] estimated that there are one and a half billion vegetarians globally. Of these, 75 million are vegetarians by choice, with this figure predicted to rise with increasing affluence and education. The remaining are vegetarians by necessity, such as those in the developing world with a lack of choices in foods that they can consume. Adoption of a vegetarian lifestyle by individuals is largely due to ethical, ecological, religious, empathy for animals, and health reasons [8,65,68,69].

In terms of pet food, ethical concerns about commercial pet food appear to be the primary motive for owners feeding cats vegetarian diets [65]. However, there have been several reports of the nutritional inadequacy of vegetarian and vegan diets for dogs and, more commonly, cats due to their obligate carnivore status, which has implications not only for health but also the welfare of both species [8,14,65,68,69,70,71].

## 5. Ingredients in Pet Food

Although a wide variety of pet foods exist, most utilise significant quantities of animal by-products, and pet food production is tightly related to both livestock production and the human food system [72]. By making use of by-product streams, the pet food industry does not directly compete with the human food industry. Instead, it reduces the environmental load of the human food system by utilising inedible meat, poultry, and fish by-products and co-products that would otherwise go to waste [73]. As a result, the transformation of low-value animal by-products into value-added pet food has played a major role in the growth and expansion of the pet food industry [74], and they are a nutritious animal-sourced ingredient for cats to meet their obligate carnivore status [52]. However, little has been reported on the palatability of individual by-product ingredients of animal origin.

### 5.1. Meat

Meat is defined as the flesh derived from any species of slaughtered mammal and is made up of muscle tissue but may also include intramuscular fat, connective tissue of the muscle sheaths and tendons, as well as blood vessels [52,59]. Lean meats lacking fat tend to have similar proportions of water and protein (75% and 25%, respectively), whether from different parts of the same carcass or even from different animals such as cattle, lamb, pigs, or poultry ([59]; Table 4).

Meats are a good source of amino acids, fat, iron, and some B vitamins such as niacin, thiamine, riboflavin, and vitamin B12 [59]. Compared to that for human consumption, meat for pet food is obtained by mechanically separating excess muscle meat from bones using a machine to deliver a final product that is finely ground and paste-like in texture [27].

### 5.2. Meat By-Products

By-products are classified as “a protein source consisting of organ meats, scrap meat, bone, blood, and fatty tissue from mammals, but do not include hair/hide, horns, hooves or teeth, or intestinal contents” [75]. Animal-sourced proteins are generally regarded as being of higher quality and superior in amino acid balance compared to other ingredients in pet food [52,76]. Additionally, the consumption of offal often reflects the feeding behaviour of the larger wild cats, which preferentially consume organ tissues [75,77]. As ingredients, offal meats are a rich source of trace elements, with levels being much higher than those in muscular tissue [78] and animal by-products, and are beneficial in providing essential nutrients.

Large differences in nutrient content are generally exhibited between different offal types, particularly in terms of the fat and vitamin contents [59,79,80].

However, both muscle and offal meats have very low calcium contents and have unfavorable calcium to phosphorus ratios that can range from 1:15 to 1:26 (Table 4). Most meat and offal are also deficient in vitamins A and D. Liver and kidney are the exceptions and provide a good source of these vitamins, although vitamin A toxicity can be a problem with the liver [59].

Although ingredients are primarily used in a blend to provide specific nutrients in diets, examining the compositional variation of individual meat by-products may help determine what drives food selection and preference on a fundamental level in companion animals.

### 5.3. Textured Vegetable Protein (TVP)

Many canned and pouched pet foods contain considerable amounts of textured vegetable protein (TVP), an extruded soybean product typically made from defatted soy grits or flour used to form meat-like chunks [81,82]. While the aim of TVP is for it to look like meat, it usually has a similar nutrient profile as soy flours [83]. Plant-based proteins used in pet food manufacturing have less complete amino acid profiles than animal-based proteins [76]. Soy is the best of the plant-based sources of protein; however, in terms of amino acids, it is rich in lysine and limited in sulfur amino acids, namely methionine and cysteine [84,85].

### 5.4. Carbohydrate Sources

Although carbohydrates are not considered essential for cats, as their natural diet contains little carbohydrate, commercial cat foods, particularly dry diets, can contain as much as 40% carbohydrates [77,86]. This is particularly evident in economy brands, in which the first ingredient is likely to be a named grain or cereal. Despite their obligate carnivore status, carbohydrates do provide a fiber source in the diet, which is important for gut health, but too much can lead to obesity [77]. Additionally, cats can utilise starch as a glucose source to provide cellular energy [77]. This does provide a cheaper source of energy for pet food manufacturers; however, proper processing or cooking is necessary to make starches digestible to cats and dogs [75]. Typical sources of carbohydrate in pet foods include various grains, such as brown rice, oats, sorghum, potatoes, and legumes [77,87,88].

## 6. Palatability and Preference

With the increasing number of pet foods available on the market, palatability is the major criterion used to measure product performance. Although interpreted in many ways, palatability is defined as the physical and chemical properties of the diet, which are linked with the promotion or suppression of feeding behaviour during the pre-absorptive period [6,26]. Rather than being associated with a want or need, palatability relates to pleasure perception or liking during consumption [89]. In short, food that is palatable is one that is seen as readily accepted by an animal [2,6,89].

### 6.1. Palatability Testing

Consumption testing (i.e., how much diet is consumed over time) is the most commonly used technique for assessing the palatability of pet foods. During the product development stages, pet food manufacturers will often use palatability studies to test product acceptance and preference. Briefly, acceptance testing is used to determine a single product’s intrinsic palatability [2], while preference testing utilises the simultaneous presentation of different diets (generally two or three diets) to determine whether one is preferred over the other(s) based on intake [2].

### 6.2. One-Bowl Test

The acceptability of food is measured using a one-bowl test, where a single product is presented to an animal [2]. This method requires the use of a defined number of animals, normally between eight and ten, and is repeated over a number of days (typically five days) as a means to eliminate environmental influences.

This method is beneficial in terms of more accurately reflecting the feeding choices provided in the home, where one product is generally presented to an animal and can be carried out using any breed and size animal. As no training is required for the one-bowl method, either kennel or pet animals can be used [6]. In addition, the cost of performing this test is relatively low. It may also help to identify a product that is completely unacceptable due to off flavors, aromas, or textures.

Although advantageous in many aspects, this one-bowl testing method is more suited for determining the acceptance of a single food, and no information on the preference or degree of liking of the food by the animals can be obtained [6]. In addition, using this method alone often does not provide enough information for marketing claims or product improvements. Finally, pet animals are likely to vary more than kennel animals in terms of the results obtained from one-bowl testing. This is likely due to the variation in prior feeding that pet animals receive. In order to minimise these effects, in-home testing should consist of animals undergoing a period where they are fed a control diet for four to five days prior to being presented with the test diet. However, this can be very time consuming, so it is not often adopted for in-house testing of diets.

### 6.3. Two-Bowl Test

The two-bowl test is the other traditional palatability testing method adopted in pet food research and involves presenting two diets simultaneously to an animal for a defined period [2]. This enables the subjects to indicate their preference for one product over another based on the quantities of food consumed during a sitting [6]. It is the most common and reliable type of test for palatability assessment studies in both cats and dogs. Two-bowl testing can be used for both kennel and pet panels, although the inability to control the testing environment in the home can result in less precise findings.

Animals are normally placed in individual testing booths, with the aim being to give them free access to food without fear of competition and to limit social interaction during the testing period [2]. These tests are normally repeated with bowl positions altered to limit the effect of side preference and evaluate the consistency of the results. Compared to cats, dogs are likely to consume both options presented, so measures of the first bowl approached, the first consumed, and the first finished are often additional observations recorded for two-bowl testing in dog studies.

The number of subjects used in the two-bowl test is also an important consideration. Ten animals assessed over five to six days or 20 animals over two or four days were used to gain 50 to 60 observations [6]. However, the use of a trained panel of eight cats for a two-hour period over five days has been frequently used as a go-to protocol [90,91,92]. Two hours for testing has proven to deliver sufficient power to consistently detect differences between diets as well as consistently reliable results through obtaining 40 subsequent measures of intake [90].

In contrast to using eight cats for five days to obtain 40 measurements, the use of more test subjects for a few days can also be adopted to provide true observations of the animals and is advantageous for revealing more quickly whether animals prefer one food over the other [6].

As highlighted by many authors, the important parameters that can be measured in the two-bowl test include [2,6,90].

- the initial choice and/or the first food product tasted
- the amount of food consumed
- the ratio of food consumed
- the percentage of food intake
- the preference ratio (quantity of food A consumed over the total amount of food distributed − food A + food B)

Two-bowl testing is beneficial when evaluating new flavor systems and product enhancements, such as comparing an experimental diet (with a new flavor) vs. a control diet (without flavor), for example [6]. It is also commonly used within the industry in the new product development stage and when comparing the food to a competitor’s product [6]. The main limitations of two-bowl testing include only being able to rank between the two foods tested, meaning only paired comparisons can be evaluated. Using this method alone could result in further paired comparisons needing to be evaluated, which can be time-consuming depending on the number of iterations necessary to be able to draw conclusions. With this in mind, two-bowl testing can tell us which food an animal prefers over another, but it does not explicitly reveal what the pet likes within the food, nor does it help identify the components or ingredients that are attractive in a food unless multiple iterations are performed [2,6].

### 6.4. Behaviour as a Measure of Palatability

As cats are not able to verbalise their likes and dislikes, studies have evaluated the behavioural response of cats to various foods as an additional objective measure of palatability [93]. A study by Van den Bos et al. [94] was able to identify certain physical responses that appeared to be related to liking or aversion to different foods, also known as taste reactivity tests. Liking towards food was distinguished by licking and sniffing the feeding bowl, licking of the lips, and grooming of their faces. However, food aversions were highlighted by the licking and sniffing of the food and the licking of the nose. These differences are quite subtle, and the difficulty in distinguishing between licking of the lips and licking of the nose has been acknowledged by Becques et al. [18] when using feeding behaviour to evaluate pet food palatability.

It is also suggested that the time cats spend sniffing food may be used to assess palatability [2]. In the study by Becques et al. [18], kibble diets of “very palatable kibble” (VPK) and “less palatable kibble” (LPK) were presented to cats for 20 h a day. These diets differed in palatability by having a super-premium poultry-based hydrolysate coating on the food in the VPK diet compared to a standard viscera-based hydrolysate coating on the LPK [2]. It was found that cats spent more time sniffing the LPK on day one and showed hesitation in consuming the diet. Furthermore, consumption of the LPK was lower than that of VPK throughout the study, indicating a preference for VPK over LPK.

In a survey conducted by Knight and Satchell [10], which compared owner-perceived palatability behaviours in cats fed vegan versus meat-based pet foods, little difference was observed in the food-oriented behaviour of cats fed conventional, raw, and vegan diets. A limitation of this survey was that the results were extremely subjective and based solely on owner-reported behaviours which are likely to show greater variability and bias compared to using a trained panel of cats that are able to discriminate foods with different sensory properties [18].

Although useful in determining which food is tastier, both consumption-based and behavioural palatability assessments are still unable to identify the specific components that drive food intake. Work is therefore required to relate food intake results from palatability studies to the nutrient and textural properties that are driving or hindering product performance.

### 6.5. Factors to Consider for Palatability Testing

Although testing methods have been established, it is also important to select suitable animal subjects to test for the palatability of foods. It is known that cats, like humans, will likely exhibit individual variation when it comes to food acceptance and preference. However, some cats can display undesirable traits, particularly “side bias”, which may severely impact palatability testing results. Side bias is common in cats and can be characterised when an animal prefers to eat from a bowl positioned on a particular side (i.e., left or right) regardless of what diet is presented [95]. Animals that exhibit this behaviour can skew results, so it is important to screen out such individuals prior to testing.

In addition to side bias, the animals’ level of hunger leading up to testing can also impact the amount of food eaten. To combat this, animals are normally fed a reduced amount of their usual food or are fasted prior to testing. Seasonal effects can also result in variability, with cats eating less in the winter than in the summer [2,25]. Therefore, it is necessary to ensure that a standard testing protocol is in place and that it is followed.

### 6.6. Palatants/Palatability Enhancers

Palatants incorporate many different macro- and micromolecules including proteins, amino acids, carbohydrates, fatty acids, peptides, vitamins, and minerals [96]. The aim of these ingredients is to enhance the sensory experience of the animal, particularly the umami T1R1 and T1R3 taste receptors, as cats are known to have a strong affinity for umami compounds [50,97]. In the pet food industry, animal protein hydrolysates have been used to create palatability enhancers via the Maillard reaction [98]. Additionally, animal proteins, as well as specific amino acids, animal fats, and emulsified meats, have been identified as important flavors that are highly palatable to cats [99].

Palatants exist in both dry and liquid forms and are commonly added to kibbles following extrusion to enhance the flavor of food [100]. In comparison, wet foods tend to have a higher palatability than dry foods due to their higher moisture content and processing techniques [101]. As a result, the inclusion levels of palatants in wet foods are generally lower than those in dry pet foods.

## 7. Palatability Drivers

While the most commonly used methods of assessing pet food palatability have been described above, limited studies have taken place to identify the dietary components that drive food intake.

### 7.1. Biological Aspects

In addition to the differences in feeding behaviour and nutrient requirements, the main factors influencing food preference in cats and dogs also vary. In dogs, odor preference has been identified as the likely driver for palatability. Hall et al. [102] presented dogs with two bowls containing one of four poultry-flavored dog diets, and in 89% of the tests, the dogs consumed more of the diet they initially selected first. Similar results were observed by Roberts et al. [103]. It was concluded that dogs were able to select their preferred diet before tasting, and it is possible that odor was a key factor in making this selection.

In kittens, preference for food is often strongly influenced by the food preferences exhibited by their mothers [15], and exposure to foods during their mother’s pregnancy via amniotic fluid and in early life can also affect a cat’s feeding behaviour [6,7,15,104]. For example, cats raised from birth on a single diet of mackerel and rice showed neophobia when offered novel foods, which contrasted with cats that were raised on a variety of foods [105]. With cats, limited exposure to different foods in early life can result in preference for that flavor, which is referred to as the primacy effect [89]. However, the primacy effect may not be observed in practice, with some cats exhibiting neophilia (i.e., the preference for a new food over a pet’s accustomed diet) when pet owners make a range of food experiences available [21,89,106]. Furthermore, when cats are presented with two foods that are both familiar and abundant, they will eat a mixture of the two to obtain a wide range of nutrients and maximise the long-term nutritional benefits [21,106].

Several factors play an important role in diet selection for cats. Cats use both smell and taste in the detection and selection of foods [23,97,107]. While not as developed as in dogs, the olfactory senses are used by cats to recognise both novel and untrusted aromas [6]. These senses are also able to detect the freshness and safety of food, which may also explain why cats display greater selectivity towards food compared with dogs [6]. When presented with chemosensory stimuli as kittens at 9 to 10 weeks and at 6 months of age, cats prefer familiar diets over unfamiliar ones [108]. It was found that cats will consume one food exclusively over another if they find the odor significantly more attractive.

Although taste and smell are both important in food selection, taste is the more dominant sense in influencing the food preference of cats as opposed to color and orthonasal olfaction [6,21,23]. As a result, more research has been published on the taste than the olfaction of cats [109].

### 7.2. Taste Receptors

Cats exhibit three groups of chemoresponsive tongue receptor units, all of which respond to different compounds. All three groups of units innervate fungiform papillae positioned in different but overlapping areas of the tongue [110]. Group I units respond to acids in general (particularly citric and malic acid), as well as certain nitrogen compounds when consumed at a neutral pH and compounds with an imidazole ring [110,111]. Group II units respond to amino acids, di- and triphosphate nucleosides, and some inorganic salts [110]. Group III unit stimuli are less well defined but are maximally sensitive to nucleotides [110,111]. In general, the sense of taste in cats is like that of other mammals, responding to salty, sour, and bitter stimuli as well as amino acids and nucleotides but showing no response to many sugars [112].

In cats, the most abundant taste receptors are those that respond to amino acids, and cats do show a preference for some amino acids [7,21,52,113,114]. An increase in spike output from geniculate ganglion chemoresponsive group II units was observed in response to L-proline, L-lysine, and L-histidine, compared to a decrease in group II discharge towards L-tryptophan and L-isoleucine [113]. Cats, therefore, appear to reject amino acids regarded as ‘bitter’ to humans such as L-arginine, L-isoleucine, L-phenylalanine, and L-tryptophan and prefer amino acids that are identified as ‘sweet’ including L-proline, L-cysteine, L-ornithine, L-lysine, L-histidine, and L-alanine [7,21,115]. Although preferring ‘sweet’ amino acids to ‘bitter’ ones (where ‘sweet’ and ‘bitter’ are defined by humans), cats do not have any functional sweet taste receptors [112], so they must have some means of differentiating between the two types of amino acids.

Generally, cats are drawn to foods with a strong umami/savory flavor, which is often related to a high concentration of amino acids, particularly L-glutamic acid [50,97]. The abundance of amino acid taste receptor units in cats is linked to meat-eating and is used to discriminate between meats of different quality [109]. This may explain why cats are known to refuse monophosphate nucleotides, which accumulate in mammalian tissue after death and provide some reasoning for their dislike of carrion [7,21].

Recent research also indicates that kokumi, described as the sensation of enhanced sweet, salty, and umami tastes, is an important taste modality for carnivores and has associations with the palatability of meat-derived compounds such as amino acids and peptides [116,117,118]. The Calcium Sensing Receptor (CaSR) has been designated as the putative kokumi taste receptor in humans and is also expressed in the circumvallate papillae of cats [116,119]. Various L-amino acids, L-amino acid derivatives, biogenic amines, glutathione (GSH) and its derivates, as well as β-aspartyl and γ-glutamyl peptides, and certain aminoglycoside antibiotics studied by Laffitte et al. [118] have been identified as agonists of the CaSR in cats. The study provides initial insight into certain components within a food that may show a direct link to palatability in cats.

### 7.3. Structural Changes to Meat Due to Age of Animal at Slaughter

For human consumption of meat, texture and meat tenderness play a vital role in consumer acceptance [120,121]. Collagen is an abundant connective tissue and contributes greatly to the variation in meat texture and tenderness [122]. Beef tenderness is lower in meat from older animals, particularly for muscles with high connective tissue strength. The bicep femoris trebled in toughness compared to the psoas major in young versus older cattle [123]. It is known that the strength and number of cross-links of intramuscular collagen increase in older animals, and the collagen becomes less heat soluble with age, therefore resulting in greater sensory toughness [121,122,124].

In the pet food industry, humanisation, defined as the circumstance in which owners consider their pet and their relationship with it as if it were human in nature, along with the premiumisation of pet foods have remained two of the most dominant trends in the market [125]. Although not studied in the pet food industry to date, the age of animal by-products included in pet food may be a factor to consider in the production of premium pet foods.

### 7.4. Palatability of Meat and Meat By-Products

While animal-sourced proteins are essential for cats, little published information exists on the relative palatability of different types of meat and meat by-products and the factors that drive preference for one meat over the other.

Studies in dogs have shown a preference for different types of raw meat, with beef being the most preferred, followed by lamb, then chicken, and horsemeat being the least preferred [126]. Preference for canned or cooked meat over fresh meat was also observed, along with minced meat over chunks of meat and canned meat over fresh meat [126]. In addition, free-ranging dogs seemingly follow the rule of thumb that “if it smells like meat, eat it” as a means of maximising the utilisation of resources that contain any quantity of protein [127,128]. However, it has also been shown that this rule of thumb is not innate and needs to be learned by pups [129].

In cats, studies have revealed preferences for fish, specifically salmon, over commercial cat food (fish, liver, chicken, or beef-flavored) and rats [130,131]. For meat products that had been diluted in distilled water, it was found that pork liver, pork kidney, tuna, and chicken were the most effective food stimuli on the tongue units, with egg white and sucrose being the least effective [132].

As well as these classical studies, preliminary research investigating the palatability of commonly used beef and lamb offal when fed raw to cats has also been reported [133]. Although the study was successful in determining the acceptance of offal, with liver being the most palatable and mechanically deboned meat (MDM) being the least palatable within beef and lamb, as well as a consistent preference for lamb over equivalent beef offal based on food intake and percentage consumption data, the underlying nutritional factors contributing to differences in palatability remain unclear.

The palatability of meat and meat by-products, both in their raw and processed forms, remains an area that is overlooked, particularly in feline nutrition studies. As the pet food industry continues to grow and utilise a wider range of animal by-products in pet food formulations, further investigation is required to understand what drives preference for certain by-products over others. Such information could be used by manufacturers to optimise ingredient inclusion levels to deliver a product that is highly palatable and cost-effective to manufacture.

### 7.5. Specific Nutrients

Nutrient components of diets, including dry matter, crude protein, crude fiber, ether extract, nitrogen free extract, ash, calcium, phosphorus, total lipids, and metabolisable energy, have been evaluated to determine their influence on palatability in the cat [97]. Using principal component analysis and linear regression, the dietary fiber content along with the mineral components calcium, phosphorus, and ash were identified as constituents that negatively affect food preference in cats.

Other than this study, limited work has been done to identify nutrient drivers and inhibitors of palatability for cats. For this reason, more research is required to bridge the gap between identifying complete foods that are more palatable and understanding the nutritional factors within them that drive intake. Modern analytical techniques such as metabolomics, which is defined by Clish [134] as the in-depth examination of metabolites in a biological specimen, may provide greater insight into the compounds responsible for characterising the nutritional and sensory properties within key ingredients in pet food [135].

### 7.6. Physical Properties of Food

Dry and wet diets are the food formats most commonly purchased by pet owners in New Zealand, but they differ significantly in their nutritional composition. Wet diets have a higher protein content, which is closer to a cat’s ‘target intake’, with more fat and minimal carbohydrates. Whereas dry foods often have less protein and similar fat levels to wet diets, and carbohydrates can be as high as 40% [104]. This may provide reasoning as to why wet food, which shows similar nutritional composition and water content as meat, may be preferentially preferred over semi-moist and dry foods, respectively [7].

#### 7.6.1. Processing

Hagen-Plantinga et al. [62] studied the effect of retort temperature on palatability using two-bowl testing. Three retort temperatures were used at different times to ensure equal lethality for the different temperatures. Higher temperatures disrupted the binding properties, negatively affecting texture and negatively affecting palatability compared to the longer processing times that were necessary for the lower temperatures.

Maillard products in wet food have a positive influence on palatability in cats [7,64], while lipid oxidation results in decreased palatability, as the off notes are easily detected by cats [7,62]. To combat this problem, antioxidants are added to pet foods to prevent the oxidation of lipids, preserve nutrient quality, and maintain product freshness [136,137,138].

#### 7.6.2. Shape and Texture

Kibbles with sharp edges are known to be unfavorable to cats, as these can cause cuts to the mouth and stomach [7]. Coating the outside of kibble with fat has a positive impact on food texture rather than contributing to flavor [7]. In terms of wet foods, stickiness and viscosity are important palatability considerations in the production of wet foods [104].

#### 7.6.3. Serving Temperature

Rejection of food by cats has been observed if served at a temperature below 15 °C or above 50 °C [7]. Additionally, if palatable foods are served chilled, refusal has also been displayed, as cats tend to prefer food at similar temperatures to the body temperature of live prey, or at least room temperature [21]. A study by Eyre and colleagues [19], which examined specific serving temperatures of wet food to older (>7 years) domestic short-haired cats, found that they preferred food served at 37 °C (i.e., body temperature) compared to room temperature of 21 °C, with food chilled to 6 °C being the least preferred. Volatile compounds were also analysed in this study, with the hypothesis that warming food may help enhance the flavor profile for aging cats and help encourage intake. Serving temperature could likely be used to create more robust feeding guidelines for older and/or fussy cats [19].

## 8. Trends in the Pet Food Industry

Finally, it is important to note that trends in the pet food industry often mimic those observed in the human food industry. Key trends that have remained throughout the years are humanisation and premiumisation, as previously described [125]. However, pet food experts believe that a movement toward greater sustainability within the industry, as well as the ability for food to deliver functional benefits, are the top trends for 2023 [139]. Sustainability not only includes exploring the use of novel insect and plant-based proteins but can be approached from other angles, such as examining the environmental sustainability effect of using more eco-friendly packaging. Although humanisation has dominated as a trend in the industry, it seems to counter the new emerging trend of sustainability.

In contrast, humanisation and the emerging trend of specialised nutrition through methods such as customising pet foods to deliver nutritional benefits seem to go hand-in-hand. This is particularly relevant as owners now view themselves more as pet parents than just pet owners [139].

These emerging trends not only show the trajectory in which pet food is heading in terms of new and novel ingredients and packaging systems, but also open the opportunity for fundamental research to take place on known ingredients that can be used to help deliver these specialised foods.

## 9. Conclusions

Pet food palatability, particularly for cats, continues to be of great importance to both manufacturers and owners. Currently, traditional palatability testing methods are used to assess the acceptance and preference of complete and balanced pet food, as well as treats. However, until very recently, few studies have used these traditional methods to assess the palatability of individual diet components, specifically meat and its by-products, which are important for the carnivorous cat [133]. It is known that cats show differences in palatability for selected by-products, however, gaps exist in our knowledge in this area, and more work is required to determine the fundamental drivers responsible for these differences. Modern techniques such as metabolomics may unlock this knowledge but are still in their infancy in pet food research. In the future, a collective approach using traditional palatability testing methods and modern analytical testing may help to not only determine the optimal inclusion level of ingredients to maximise palatability but also the nutrients responsible for driving preference, which, to date, has been understudied at the fundamental level.

## Figures and Tables

**Table 1:** The protein and 11 essential amino acids listed for cats and 10 essential amino acids listed for dogs are required for adult maintenance (adapted from [27]).

Requirement on DM Basis (%)	Adult Maintenance			
	Cat		Dog	
	Minimum	Maximum	Minimum	Maximum
Crude Protein	26	-	18	-
Essential amino acids				
Taurine (canned/extruded)	0.2/0.1	-	No requirement	-
Arginine	1.04	-	0.51	-
Histidine	0.31	-	0.19	-
Isoleucine	0.52	-	0.38	-
Leucine	1.24	-	0.68	-
Lysine	0.83	-	0.63	-
Methionine	0.20	1.5	0.33	-
Phenylalanine	0.42	-	0.45	-
Threonine	0.73	-	0.48	-
Tryptophan	0.16	1.7	0.16	-
Valine	0.62	-	0.49	-

**Table 2:** Vitamin and mineral requirements in cats and dogs for maintenance (adapted from [27]).

Nutrient	Units DM Basis	Cat		Dog	
		Minimum	Maximum	Minimum	Maximum
**Minerals**					
Calcium	%	0.6		0.5	2.5
Phosphorus	%	0.5		0.4	1.6
Ca:P ratio				1:1	1:2
Potassium	%	0.6		0.6	
Sodium	%	0.2		0.08	
Chloride	%	0.3		0.12	
Magnesium	%	0.04		0.06	
Iron	mg/kg	80		40	
Copper	mg/kg	5		7.3	
Manganese	mg/kg	7.6		5.0	
Zinc	mg/kg	75		80	
Iodine	mg/kg	0.6	9.0	1.0	11
Selenium	mg/kg	0.3		0.35	2
**Vitamins and others**					
Vitamin A	IU/kg	3332	333,300	5000	250,000
Vitamin D	IU/kg	280	30,080	500	3000
Vitamin E	IU/kg	40		50	
Vitamin K	mg/kg	0.1		-	
Thiamine	mg/kg	5.6		2.25	
Riboflavin	mg/kg	4.0		5.2	
Pantothenic acid	mg/kg	5.75		12	
Niacin	mg/kg	60		13.6	
Pyridoxine	mg/kg	4.0		1.5	
Folic acid	mg/kg	0.8		0.216	
Biotin	mg/kg	0.07		-	
Vitamin B12	mg/kg	0.020		0.028	
Choline	mg/kg	2400		1360	

**Table 3:** Macronutrient contents of dry, semi-moist, and canned dog foods on an as-fed or dry matter basis (adapted from [52]).

	Dry		Semi-Moist		Wet	
	As-Fed	Dry Matter	As-Fed	Dry Matter	As-Fed	Dry Matter
Moisture (%)	6–10	0	15–30	0	75	0
Fat (%)	7–20	8–22	7–10	8–14	5–8	20–32
Protein (%)	16–30	18–32	17–20	20–28	7–13	28–50
Carbohydrate (%)	41–70	46–74	40–60	58–72	4–13	18–57
ME (kcal.kg^−1^)	2800–4050	3000–4500	2550–2880	3000–4000	875–1250	3500–5000

**Table 4:** Typical nutrient content of selected meat and meat by-products (adapted from [59]).

	Water g/100 g	Protein g/100 g	Fat g/100 g	Calcium g/100 g	Phosphorus g/100 g	Energy kcal/100 g
Raw lean meats						
Pork	71.5	20.6	7.1	0.008	0.20	147
Beef	74.0	20.3	4.6	0.007	0.18	123
Veal	74.9	21.1	2.7	0.008	0.26	109
Lamb	70.1	20.8	8.8	0.007	0.19	162
Chicken	74.4	20.6	4.3	0.01	0.20	121
**Average**	**73.0**	**20.7**	**5.5**	**0.008**	**0.20**	**132**
Offals						
Fatty lungs	73.1	17.2	5.0	0.01	0.19	114
Heart	70.1	14.3	15.5	0.02	0.18	197
Heart (trimmed)	76.3	18.9	3.6	0.005	0.23	108
Liver (fresh)	68.6	21.1	7.8	0.001	0.36	163
Green tripe	76.2	12.3	11.6	0.01	0.10	154
Dressed tripe	88.0	9.0	3.0	0.08	0.04	63
Sheep lungs	76.0	16.9	3.2	0.01	0.20	96
Beef kidney	79.8	15.7	2.6	0.02	0.25	86
**Average**	**76.0**	**15.7**	**6.5**	**0.020**	**0.19**	**123**

## Data Availability

No new data were created or analyzed in this study. Data sharing is not applicable to this article.

## Abbreviations

AAFCO, Association of American Feed Control Officials; aw, water activity; Ca, calcium; CaSR, calcium sensing receptor; CKD, chronic kidney disease; DHA, docosahexaenoic acid; DM, dry matter; EPA, eicosapentaenoic acid; F0, lethality value; LPK, less palatable kibble; MDM, mechanically deboned meat; NAD, nicotinamide adenine dinucleotide; NADP, nicotinamide adenine dinucleotide phosphate; P, phosphorus; TVP, textured vegetable protein; VPK, very palatable kibble.
